# A real-world study of foreign body aspiration in children with 4227 cases in Western China

**DOI:** 10.1038/s41598-024-65876-7

**Published:** 2024-07-02

**Authors:** Quan Wang, Xiangpan Kong, Gang Wang, Jiangtao Dai, Yonggang Li, Chun Wu, Zhengxia Pan, Ling He, Hongbo Li

**Affiliations:** 1 Department of Cardiothoracic Surgery, Ministry of Education Key Laboratory of Child Development and Disorders; International Science and Technology Cooperation Base of Child Development and Critical Disorders; National Clinical Research Center for Child Health and Disorders, Chongqing Key Laboratory of Pediatrics, Chongqing Key Laboratory of Children Urogenital Development and Tissue Engineering, Chongqing Higher Institution Engineering Research Center of Children’s Medical Big Data Intelligent Application, Children’s Hospital of Chongqing Medical University, No.136 Zhongshan Second Road, Yuzhong District, Chongqing, 400014 China; 2 Department of Urology, Children’s Hospital of Chongqing Medical University, Chongqing, China; 3 Department of Radiology, Children’s Hospital of Chongqing Medical University, Chongqing, China

**Keywords:** Foreign body aspiration, Rigid bronchoscopy, Clinical characteristics, Epidemiology, Outcomes research, Paediatric research

## Abstract

The early diagnosis and treatment of foreign body aspiration (FBA) can significantly improve the overall prognosis of children. There are significant differences in the epidemiology and clinical characteristics of FBA in different regions. Therefore, we conducted a real-world study in the western region of China with over 4000 patients. The aim of this study was to improve the understanding of FBA in terms of its types, the specific months of its occurrence, and the distribution of primary caregiver characteristics in western China. We collected the clinical and epidemiological data of children who were diagnosed with FBA in our hospital over the past 20 years through a big data centre. We matched the data of healthy children who underwent routine physical examinations at the paediatric health clinic during the same period to analyse the differences in the data of actual guardians. A total of 4227 patients from five provinces were included in this study. Foreign bodies were removed by rigid bronchoscopy in 99.4% (4202/4227) of patients, with a median age of 19 months and a median surgical duration 16 min. January was the most common month of onset for 1725 patients, followed by February, with 1027 patients. The most common types of foreign objects were melon peanuts, seeds and walnuts, accounting for 47.2%, 15.3%, and 10.2%, respectively. In the FBA group, the proportion of grandparents who were primary caregivers was 70.33% (2973/4227), which was significantly greater than the 63.05% in the healthy group (2665/4227) (P < 0.01). FBA most commonly occurs in January and February. More than 60% of FBAs occur between the ages of 1 and 2 years, and the incidence of FBA may be greater in children who are cared for by grandparents. A rigid bronchoscope can be used to remove most aspirated foreign bodies in a median of 16 min.

## Introduction

Foreign body aspiration (FBA) in children is not rare and can be fatal if the diagnosis is delayed^1^. Difficulty of clearly observation on aspiration event led to a considerable proportion of initial misdiagnosis^2^. For children with unclear diagnoses, doctors should identify risks as early as possible and conduct bronchoscopy examination in a timely manner even if there is no evidence on imaging examinations^3^. However, for most doctors, making appropriate clinical decisions is difficult due to a lack of experience.

Management of FBA is also challenging, and both the presence of residual foreign bodies and a prolonged surgical duration increase the risk of FBA in patients^4^. The academic community has not yet reached a consensus on the optimal method^5^. The characteristics of FBA vary significantly in different regions, and few studies in developing countries have been reported^6^.

Regional children’s hospitals were established for historical reasons. Our centre is likely the only institution within 100,000 square kilometres that is equipped to treat FBA and has treated more than 5,000 patients using rigid bronchoscopy in the past twenty years. We hope that this large sample size study can help more doctors understand the diagnosis and treatment of FBA, especially in developing countries.

Furthermore, in this study, we focused on a series of epidemiological characteristics that were previously ignored, including the season of onset and differences in guardianship, to help the academic community more comprehensively understand and prevent FBA.

## Methods

### Study design, settings, and participants

This retrospective observational study was authorized by the ethics committee of Children's Hospital of Chongqing Medical University (approval number: 2020–33). All patient guardians signed authorization forms at admission, allowing doctors to use the patient's clinical data. This study was conducted in accordance with the Helsinki Declaration^7^ and did not involve any biological samples. This study was designed and reported in accordance with the reporting of observational studies in epidemiology statement (STROBE)^8^.

The data were collected through the big data system of the Children's Hospital of Chongqing Medical University Children's Hospital^9^ from Jan. 2000 to Jan. 2020. Patients diagnosed with bronchial or tracheal foreign bodies via bronchoscopy in the Department of Cardiothoracic Surgery were excluded. Children with laryngeal foreign bodies were not included. Any children with incomplete data were excluded from this study.

The data of children who underwent routine child health checks and showed no abnormalities were acquired through a big data platform. These data were automatically matched one-on-one according to the age, sex, and month of diagnosis of patients with FBA. We will only extract detailed information about the guardians of these normal children for analysis.

All patients underwent routine medical history collection and physical examination. For patients with stable conditions and no clear history of FBA, we recommend computed tomography (CT) scanning.

### Bronchoscopy procedure and postoperative treatment

Suspicious cases will be undergone on rigid bronchoscopy anyway. For any child with unstable vital signs, rigid bronchoscopy was performed. Otherwise, we waited to perform bronchoscopy examination at night. For patients whose foreign bodies could not be removed via rigid bronchoscopy, flexible bronchoscopy or thoracotomy was performed based on the patient's condition. For children with serious complications, such as severe pneumonia bronchopneumonia, larynx oedema, and lung abscess empyema, we recommended treatment and chose an appropriate time for discharge. Otherwise, the patient was discharged the day after the foreign body was removed.

### Variables and data sources measurement

In assessment of major caregivers, if anyone of parents does not work and takes care of their child at home, we define it as “parents”. If both parents of the children work full-time and the main caregivers are grandparents, we define it as “grandparents”. Other situations, such as hiring a full-time nanny or other family members to take care of the children, are defined as “others”. The main caregivers for all patients in our hospital were classified upon admission, and all classifications were recorded in the electronic system. The location of the foreign body was determined based on the bronchoscopy results. We located the primary foreign body in cases of multisite bronchial foreign bodies.

The presence of pulmonary consolidation and atelectasis, obstructive emphysema, and mediastinal oscillation during inhalation on X-ray images indicate the presence of foreign objects. On CT examination, abnormal density shadows or discontinuities in the airway suggest the presence of foreign bodies. After all the authors confirmed the search conditions, all the data were extracted from electronic information systems through big data platforms. Two independent authors organized the data based on the data extraction tables. After review by a third author, any discrepancies in the data were submitted to all the authors for discussion and review before confirmation.

Complications included pneumonia bronchopneumonia, atelectasis, lung abscess empyema, larynx oedema, bronchiectasis pneumothorax pneumomediastinum, tracheal laceration cardiopulmonary arrest and other infections.

### *Bias*, study size and quantitative variables

This study lasted for 20 years, and different doctors may have had different years of diagnostic and treatment experience; additionally, there may be some internal heterogeneity among all patients. All the data were obtained from our hospital's big data platform. Although the data on the big data platform have been verified several times, we still cannot completely avoid potential errors. This study does not involve any intergroup comparisons; therefore, there is no clear sample size limit. The quantitative variables used in this study included age and duration of surgery. Age grouping was based on epidemiological data of previous cases of FBA. We reported these quantitative variables directly without any conversion, such as logarithmic conversion.

### Statistical methods

Continuous variables with an abnormal distribution are expressed as the median with interquartile interval (IQR). The distribution rates were compared using the chi-square test with SPSS version 24 (IBM, Armonk, NY, USA). We provide descriptive information for most of the data. Visualization of the data was achieved using R (http://www.R-project.org).

### Ethics approval and consent to participate

This retrospective observational study was authorized by the ethics committee of Children's Hospital of Chongqing Medical University (Approval Number: 2020–33). All patient guardians had signed authorization forms in admission, authorizing doctors to use the patient's clinical data as a routine requirement in our hospital. This study strictly adheres to the Helsinki Declaration and does not involve any biological samples.

### Consent for publication

All authors agree to publish.

## Results

**Figure 1:**
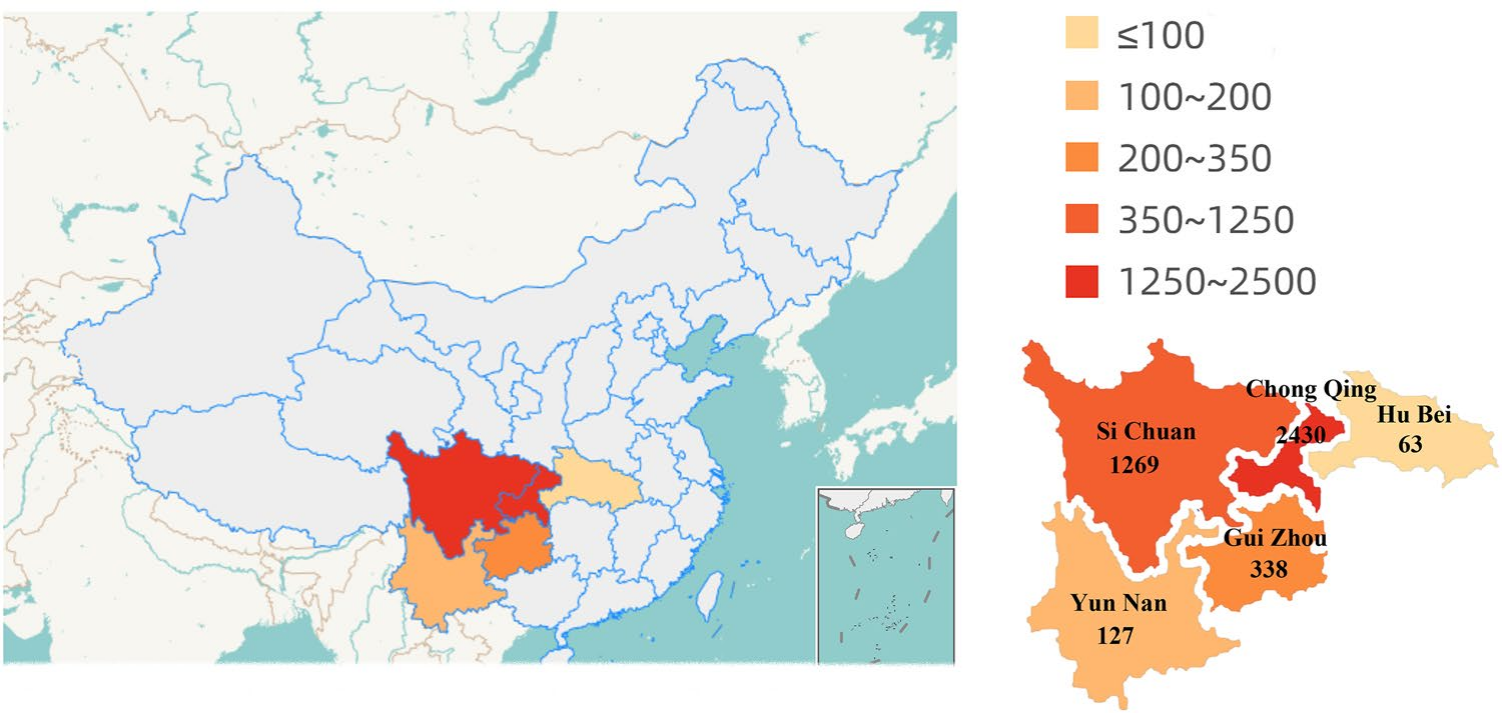
Distribution of patients with foreign body aspiration in different regions.

**Figure 2:**
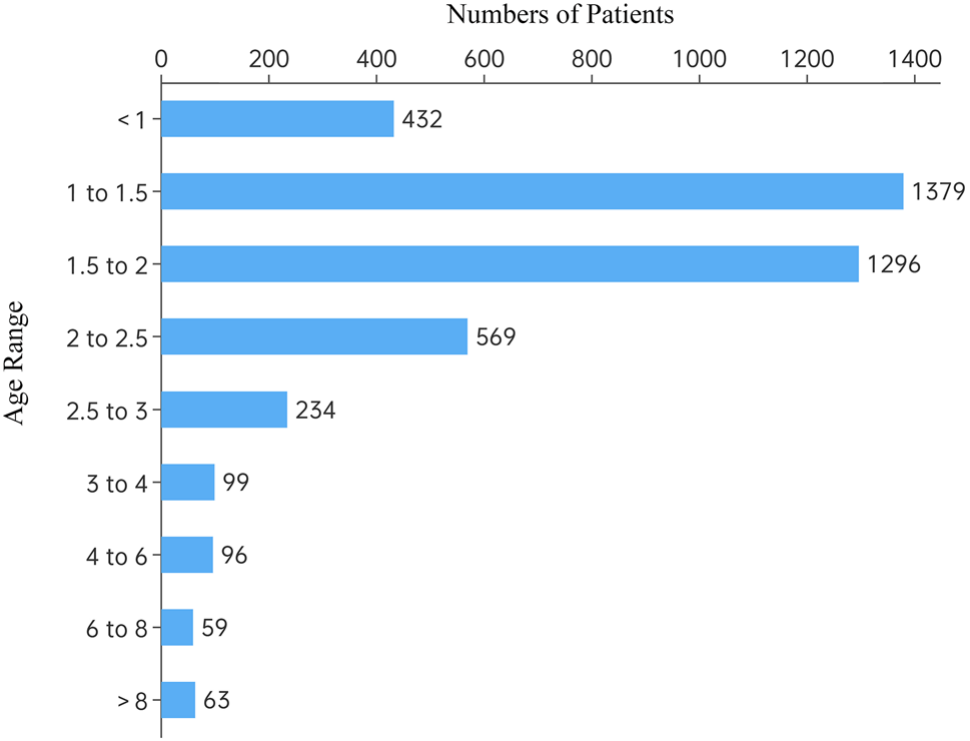
Age distribution of patients with foreign body aspiration.

A total of 4227 patients were included in this study, including 2776 boys and 1460 girls. This study included patients from five provinces, namely, Chongqing, Sichuan, Guizhou, Hubei, and Yunnan. The details of the distribution are shown in Fig. 1, and approximately half of the children were from Chongqing. The median age of the patients was 19 months (IQR 14 to 24 months), and 2675 (63.28%) of the children were between the ages of 1 and 2 years. Less than 10% of patients were over 3 years old (317/4227, 7.5%) (Fig. 2). Detailed clinical data are presented in Table 1.

**Table 1:** Demographic and clinical characteristics.

Variables	Numbers (4227)	Proportion (%)
Sex		
Male	2767	65.46
Female	1460	35.54
Region		
Chongqing	2430	57.49
Sichuan	1269	30.02
Guizhou	338	8
Yunnan	127	3
Hubei	63	1.49
Major caregiver		
Parents	1056	24.98
Grandparents	2975	70.38
Others	198	4.64
Diagnosis time		
Less than 24 h	2306	54.55
More than 24 h	1921	45.45
First hospital		
Yes	569	13.46
No	3658	86.54
Clear diagnosis outside we hospital		
Yes	2773	65.6
No	1454	34.4
Anti infection treatment outside we hospital		
Yes	1426	33.74
No	2801	66.26
Elevated white blood cells		
Yes	2975	70.31
No	1252	29.69
Suspected history of inhalation		
Yes	2665	63.05
No	1562	36.95
Imaging diagnosis		
X-ray	1184	28.01
CT scan	2815	66.6
No evidence	123	2.91
Not performed	105	2.48
Complication		
Yes	1748	41.35
No	2479	58.65
Remove foreign body by rigid bronchoscopy		
Yes	4203	99.43
No	25	0.57
Duration of surgery (min)	16 (12–22)	

**Figure 3:**
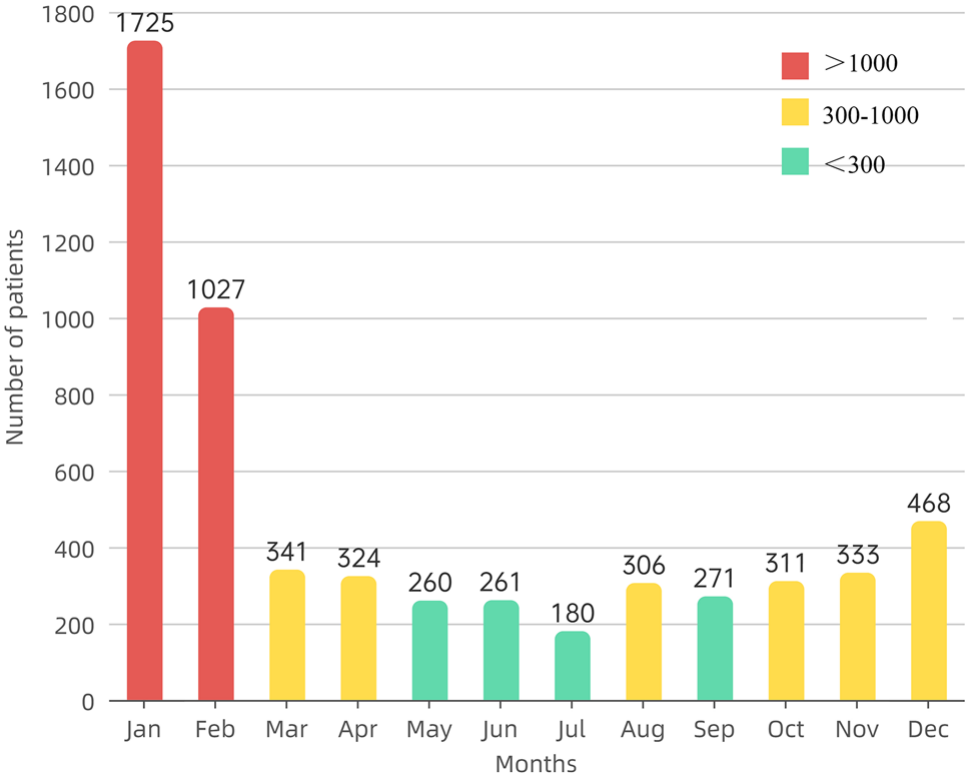
The onset time of foreign body aspiration in patients.

The most common months of onset for patients were January and February, with 1725 and 1027 children, respectively. A total of 468 children were diagnosed with FBA in December. The least common month of onset for patients was July, with only 180 children diagnosed. The number of patients diagnosed with FBA in the remaining eight months ranged between 250 and 350 (Fig. 3).

A total of 3658 patients (86.54%) did not visit our hospital first; 2773 children were suspected of having bronchial foreign bodies in other local hospitals, but the hospitals were not equipped to treat the patients. In this study, 2665 children (63.05%) had a history of suspected FBA. However, only 961 families of the affected children claimed to have witnessed the moment of foreign object inhalation with their own eyes or saw the specific scene of inhalation through surveillance playback. The most common clinical manifestation was cough, which occurred in 2903 patients (68.68%). A total of 1224 children with varying degrees of dyspnoea were reported.

**Figure 4:**
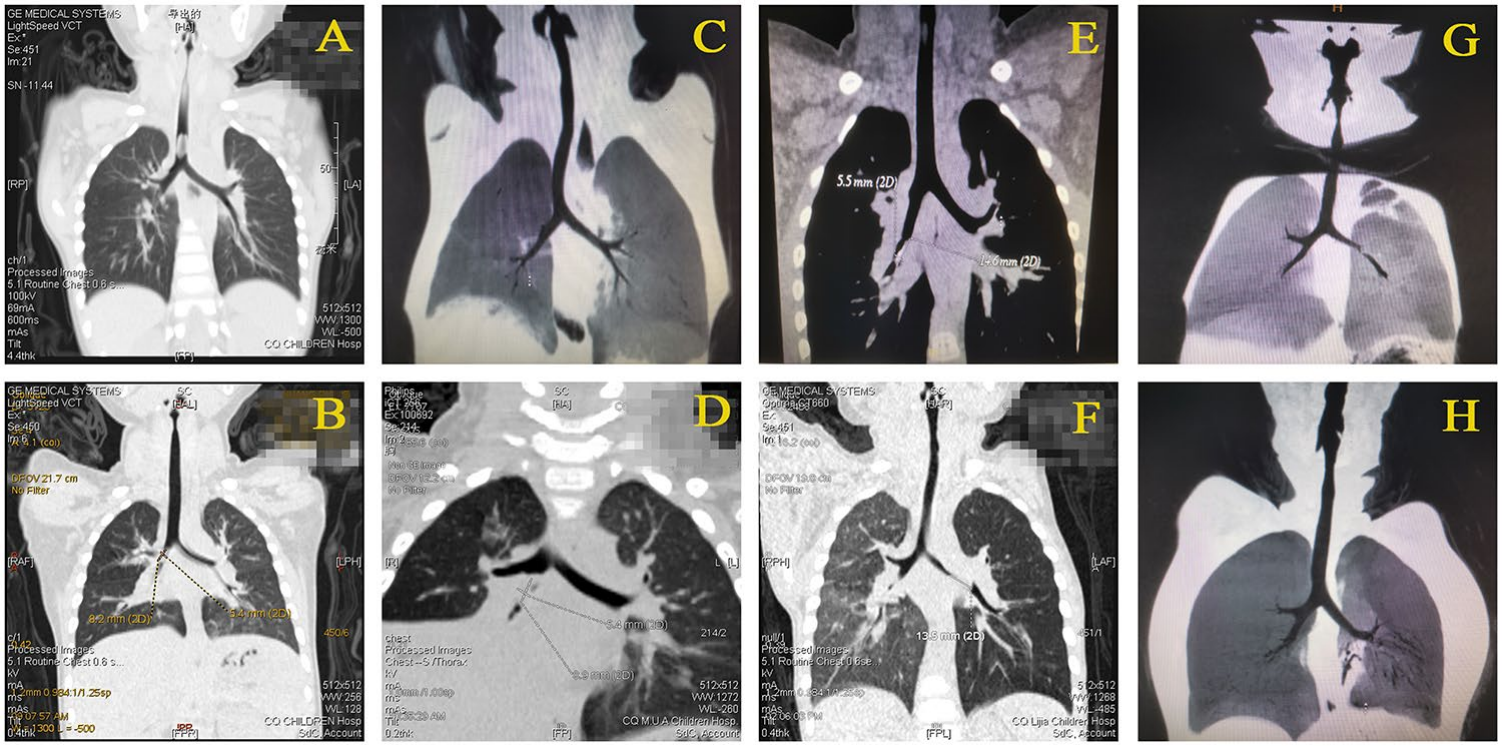
CT images of foreign bodies. **A** trachea; **B** right main bronchus; **C** right superior lobar bronchus; **D** right middle lobar bronchus; **E** right inferior lobar bronchus; **F** left main bronchus; **G** left superior lobar bronchus; **H** left inferior lobar bronchus.

Evidence of foreign objects was observed on the X-ray images of 1184 (28.01%) patients. A total of 2815 (66.60%) children did not undergo X-ray examination or did not have obvious evidence of foreign objects on X-ray examination, so CT examination was used to determine the presence of foreign objects (Fig. 4). A total of 105 patients underwent rigid bronchoscopy due to their urgent condition without any imaging examinations. No evidence of bronchial foreign bodies on X-ray or CT image was observed in 123 patients. The imaging doctor did not even observe any signs of uneven lung inflation in these 123 patients.

All patients underwent rigid bronchoscopy to remove foreign bodies, and the average median surgical duration was 16 min (IQR 12–22 min). The foreign bodies of 25 patients were not removed via rigid bronchoscopy. Among them, in 11 patients, flexible bronchoscopy was performed to remove foreign bodies because the foreign body was in the tertiary bronchi, thereby limiting the movement of bronchoscope. Seven of the 11 patients had foreign bodies located in the secondary bronchus; however, the surgeon caused the foreign bodies to progress deeper during the removal process, making its smooth removal difficult. Fourteen patients underwent thoracotomy to remove foreign bodies due to prolonged inhalation of foreign objects and significant granulation hyperplasia, and abnormal bleeding occurred during the process of removing the rigid bronchoscope. Complications were observed in 1748 patients (41.35%). Unfortunately, five children with FBA in the trachea died in the end. Two patients experienced severe breathing difficulties at admission, and we used the Heimlich emergency treatment but were unable to expel the foreign body. We were able to remove the foreign body using a rigid bronchoscope in the operating room in less than 15 min. Two patients experienced severe hypoxia during surgery. The foreign body was smooth, metal, and difficult to remove. We attempted to push the foreign body into one side of the bronchus but were unsuccessful. The foreign body was finally removed in 25–27 min. A child experienced an anaesthetic accident, which resulted in cardiac arrest. All five of these children suffered severe brain damage, and the family eventually gave up.

The most common locations of foreign bodies were the right main bronchus (45.0%), left main bronchus (28.8%) and trachea. (8.1%). The proportions of main foreign bodies in the right and left bronchi were 52.2% and 39.7%, respectively. Approximately 4% (186/4227) of foreign bodies were in multiple bronchi and their secondary structures. Such cases are relatively complex, and we focus on the location of the main foreign object.

**Figure 5:**
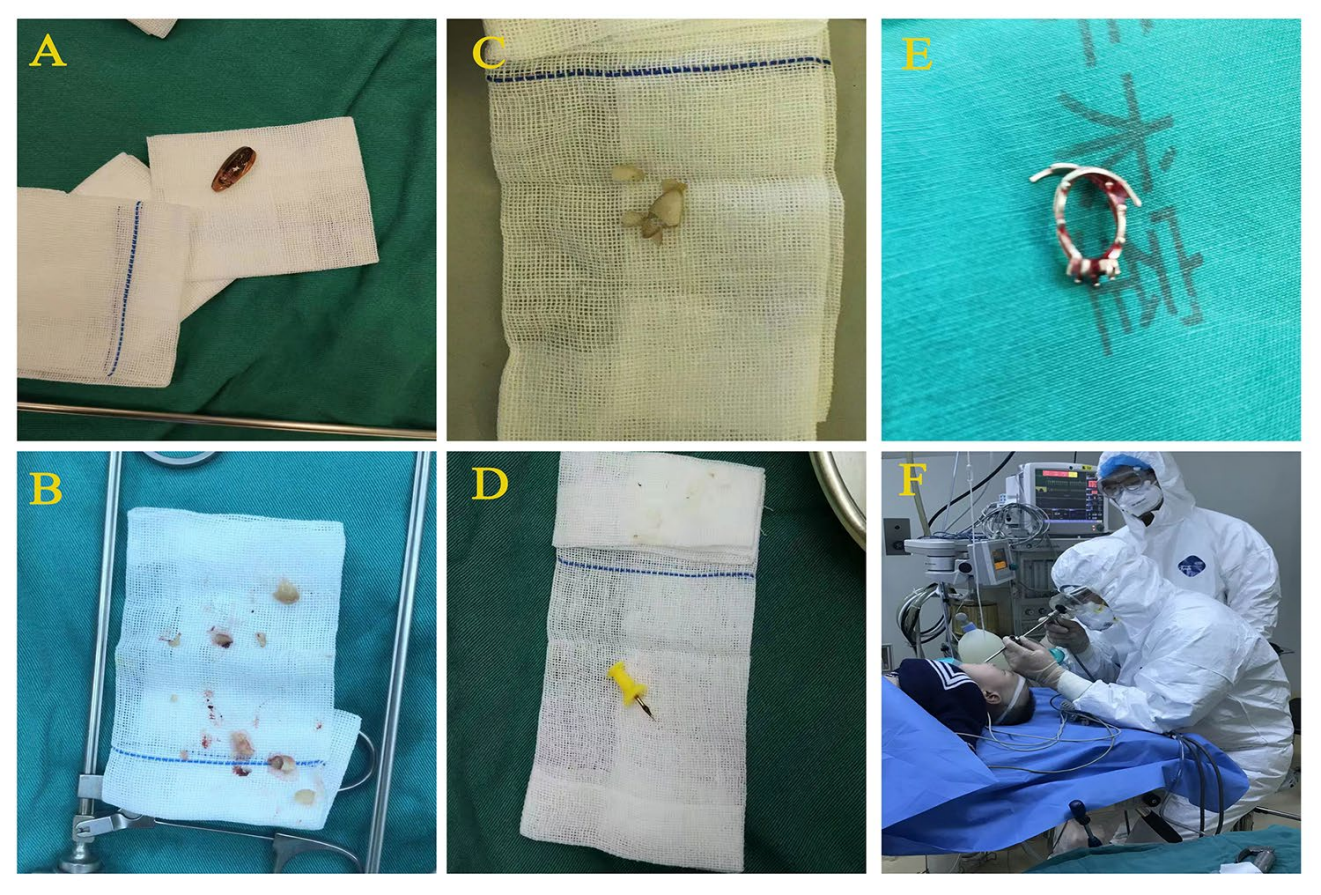
Foreign objects removed by rigid bronchoscopy. **A** Seeds; **B** peanuts; **C** almond; **D** thumbtack; **E** ring; **F** remove foreign bodies for patients with COVID-19 in 2020.

The most common types of foreign objects are melon peanuts, seeds and walnuts, accounting for 47.2%, 15.3%, and 10.2%, respectively. In comparison, metals and plastics accounted for less than 5% (Fig. 5).

Finally, we compared the actual caregivers of patients with FBA and those of normal control patients. In the FBA group, the proportion of grandparents was 70.33% (2973/4227), which was significantly greater than the 63.05% in the healthy group (2665/4227) (P < 0.01). Among the group of grandparents, the patients had a clearer history of FBA and a faster diagnostic time (Table 2). This suggests that children cared for by grandparents are more prone to have bronchial foreign bodies mainly because grandparents are more inclined to give children dangerous items such as melon seeds or peanuts and are more likely to neglect the inhalation of foreign objects, thereby delaying diagnosis.

**Table 2:** Demographic and clinical characteristics in different subgroups of caregivers.

Variables	Groups	Parents (1056)	Grandparents (2973)	Others (198)	P value
Age (months) Median (IQR)		19.0 (14.0,24.0)	19.0 (14.0,24.0)	18.0 (14.0,24.0)	0.720
Sex N (%)	Male	374 (35.42)	1013 (34.07)	73 (36.87)	0.571
	Female	682 (64.58)	1960 (65.93)	125 (63.13)	
Diagnosis time N (%)	Less than 24 h	426 (40.34)	1764 (59.33)	116 (58.59)	< 0.001
	More than 24 h	630 (59.66)	1209 (40.67)	82 (41.41)	
First hospital N (%)	No	794 (75.19)	2694 (90.62)	170 (85.86)	< 0.001
	Yes	262 (24.81)	279 (9.38)	28 (14.14)	
Suspected history of inhalation N (%)	No	629 (59.56)	867 (29.16)	66 (33.33)	< 0.001
	Yes	427 (40.44)	2106 (70.84)	132 (66.67)	
Complication, N (%)	No	630 (59.66)	1727 (58.09)	122 (61.62)	0.461
	Yes	426 (40.34)	1246 (41.91)	76 (38.38)	
Clear diagnosis outside we hospital N (%)	No	667 (63.16)	721 (24.25)	66 (33.33)	< 0.001
	Yes	389 (36.84)	2252 (75.75)	132 (66.67)	

## Discussion

This large sample study yielded valuable insights into the epidemiology of FBA in the western region of China. Research shows that FBA is more common in January and February, with most cases occurring in children aged 1–2 years, especially those cared for by grandparents. Rigid bronchoscopy is effective in treating FBA, and the median duration of the procedure is 16 min.

Epidemiology can help us understand FBA and try to prevent children from inhaling foreign objects. Three-quarters of affected children are under the age of 3^10^, but in our study, this proportion exceeded 90%. More than 60% of the children were between the ages of 1 and 2 years. The difference between our research and previous reports may be due to the different distributions of foreign object types^11^. Inorganic FBs, especially magnets, are not rare in high-income countries^12^. However, more than four-fifths of the foreign objects in our study were nuts, seeds, and beans. Metal and plastic only accounted for less than 5%. This may be related to the dietary habits and economic level of the western region of China. In summary, for children in this age group, the lack of coordination in oropharyngeal function and the start of autonomous walking significantly increased their risk of FBA, which also explains why the proportion of boys is significantly greater^13^.

The distribution of the months of onset seems to confirm our above speculation. More than 60% of patients develop symptoms in January or February, which is also the month of the Chinese New Year. During the Spring Festival, families usually consume large amounts of melon seeds and peanuts. Moreover, FBA may be more common on specific days of the week, and children may be more likely to encounter risky objects such as melon seeds and peanuts during weekend dinners. Further research is needed to confirm this hypothesis in the future.

Another noteworthy finding is that a greater proportion of patients with FBA are under the care of their grandparents. The specific reason is not yet clear. We speculate that this may be due to grandparents having less understanding of the risk of FBA and being more inclined to allow children to eat nuts. We may need to provide education to the grandparents of children aged 1–2 years to help them better understand the related risks, especially during the Spring Festival holiday. Providing nuts to children aged 1–2 years, especially when they are not quiet, carries a certain risk based on our experience. Conventional feeding can meet the various nutritional needs of children and does not require additional supplementation through this method. Notably, in our study, children who were taken care of by grandparents had a clearer history of foreign body inhalation and had a shorter diagnostic time. This indicates that grandparents closely monitor their grandchildren, and the higher incidence of FBA may only be due to insufficient awareness of related risks.

Obviously, the most important thing for FBA is early diagnosis. The diagnosis is delayed in approximately 40% of patients, thereby potentially increasing the risk of mortality^12^. Observation of aspirated objects is likely the strongest evidence for diagnosing FBA^14^. In this study, 63% of patients had a history of suspected FBA, but less than 30% of the caregivers of the patients confirmed the presence of FBA at the first consultation. Some children's actual guardians may avoid this issue due to concerns about being criticized by other family members. With the popularization of home monitoring devices in our region, the proportion of patients with a clear history of FBA may increase.

For patients without a history of FBA, clinical manifestations often lack specificity. These patients may have symptoms such as coughing, hoarseness and wheezing. Auscultation can reveal asymmetric break sounds^15^. Moreover, some children may have no symptoms or signs in the early stages, followed by fever and elevated white blood cell count. These patients may receive active anti-infection treatment outside the hospital while ignoring the possibility of bronchial foreign bodies^16^. For these patients, imaging has important value, especially high-resolution CT scans. With existing technology, CT can easily reveal abnormal density shadows in the airways, and 3D reconstruction can reveal airway discontinuity. At the same time, we are more likely to see indirect signs such as uneven inflation and obstructive emphysema. In our study, less than 3% of children had no direct or indirect evidence on CT scans, with the vast majority being from before 2010.

However, we must emphasize that for children with a clear history of FBA or unstable vital signs, bronchoscopy is the most appropriate diagnostic and treatment method. Imaging examination is not mandatory for FBA. The details of the clinical pathway for the management of FBA can be found in a previous study^3^.

Rigid bronchoscopy is the most common treatment method for FBA^17^. This procedure allows clear visibility of various types of foreign objects and maintains good airway ventilation. The main problem associated with rigid bronchoscopy is that it can be challenging when the objects are aspirated into deeper bronchial trees^18^. In tertiary bronchi, the rigid bronchoscope is limited in terms of manoeuvrability and is difficult to adjust to the appropriate angle, which may increase the risk of injury. Some doctors tend to use a flexible bronchoscope so as to achieve more thorough removal of foreign bodies, as the flexible bronchoscope can better remove foreign bodies located in thinner bronchial trees^19^. In our experience, the rigid bronchoscope is suitable for the removal of most aspirated foreign objects because there is no need to connect any display devices, and there have not been many reports of residual foreign bodies in the distal bronchi. FBA is usually an emergency, so a rigid bronchoscope for faster and safer removal of foreign bodies may be more in accordance with clinical practice requirements. The average surgical duration was approximately 17 min. This still depends on the doctor's habits. Otolaryngologists and thoracic surgeons may be more inclined to use a rigid bronchoscope, while respiratory physicians prefer a flexible bronchoscope. Foreign bodies that cannot be removed by rigid bronchoscopy require flexible bronchoscopy or surgery, and in our centre, this proportion is approximately 0.5%.

Foreign bodies in the right bronchus are considered more common because the right bronchus has a steeper shape and a smaller angle to the midline of the lower edge of the trachea, making it easier for the foreign body to become stuck in this position^20^. However, according to our data, the proportion of foreign bodies in the right bronchus accounts for only approximately 50% of all foreign bodies in the airway.

There are still some limitations in the study. First, there are regional restrictions. Since FBA is mainly considered an emergency, the patients in our institution came from five provinces, which may not fully represent the situation in western China. Second, most patients included in this study underwent rigid bronchoscopy, so it is not possible to accurately compare the therapeutic differences between rigid bronchoscopy and flexible bronchoscopy. However, to our knowledge, this is currently the largest sample single centre study. We hope that this study will help more people understand the epidemiology, diagnosis, and treatment of bronchial foreign bodies in developing countries.

## Conclusion

FBA most commonly occurs in January and February. More than 60% of FBA occur between the ages of 1 and 2 years, and the incidence of FBA may be higher in children who are cared for by grandparents. A rigid bronchoscope can be used to remove most aspirated foreign bodies within a median of 16 min.

## Data Availability

All the data of this study can be disclosed and shared, the datasets used and/or analyzed during the current study available from the corresponding author on reasonable request.
